# AI impacts on supply chain performance : a manufacturing use case study

**DOI:** 10.1007/s44163-023-00061-9

**Published:** 2023-05-04

**Authors:** Stefan Walter

**Affiliations:** VTT Technical Research Centre of Finland, Espoo, Finland

**Keywords:** Digitalisation, Artificial intelligence, Supply chain, Manufacturing, Cognitive technologies, IT architecture

## Abstract

The integration of cross-company activities to form global supply chains (SC) has several benefits, including reducing costs, minimizing energy and resource waste, and promoting relationships for improving all network actors. However, as the number of tiers of suppliers and customers increases, monitoring processes and identifying problems becomes more challenging, which can threaten the continuity of the SC. To address this issue, the EU knowlEdge project proposes using artificial intelligence (AI) solutions that are distributed, scalable, and collaborative to enable automatic monitoring and learning in the SC. This approach replaces rigid organization with flexible networks that leverage self-learning algorithms and automatic value creation, thereby facilitating knowledge sharing. The project unifies technologies from various domains, including AI, data analytics, edge, and cloud computing, into a software architecture that offers a systemic solution rather than an incremental improvement. This architecture enhances SC performance, including adaptability and autonomy, and enables industry to adopt adaptive strategies. The platform’s functionality is tested in manufacturing, where it will improve production monitoring and planning and enable human intervention and learning. The AI application is expected to increase performance on various business and production indicators, which will also have an impact beyond the factory floor. With this approach, managers can respond quickly to changing customer requirements, while deviations in planned processes can be addressed more effectively. Additionally, the research conducted by the project will provide insights into future management and learning in SC.

## Introduction

In today's global economy, companies and their operations are virtually inseparable from their supply chains [1, 2]. Due to the integrative nature of supply chains, they are often also referred to as value chains or value networks. While this is originally an allusion to Porter’s value chain concept, whereby an organisation is a sequence of value-adding activities, it also recognises the value creation potential that arises from the sometimes far-reaching and global spread of production activities [1, 3, 4]. The extension and outsourcing of activities are, of course, based on the recognition that different organisations specialise, leading to product differentiation and relative cost advantages. Their reintegration in the form of global supply chains creates real competitive advantages so that competitiveness is now a question of how best to organise supply chains [5–8].

In addition to the usual performance targets within the supply chain, which focus primarily on product and process improvements, and in logistics, for example, on performance levels and quality, other targets such as those concerned with ecological efficiency and avoidance of waste have received great attention [9–15]. Thus, processes surrounding modern supply chains are based on a pronounced dynamism, complexity and lack of visibility. Consequently, supply chain management is constantly occupied with reducing complexity wherever possible and creating transparency [16–18].

The error-free execution of production and logistics processes characterises a form of robustness. Deviations from targets or plans within a certain range can be eliminated independently. Many factors, some of which can be found in the design of a supply chain, others in areas such as production or distribution, have an influence on the quality leadership that is so important for competitiveness. These factors include, for example, an increase in complexity due to network expansion, a lack of coordination between areas or partners in a supply chain, process disruptions, delayed or erroneous information flows and insufficient service levels and the associated loss of customers [4]. Where robustness and resilience, which refers to the ability to recover quickly and effectively from disruption, are to be consolidated or even increased, sacrifices often have to be made in terms of financial gain [19]. However, recent developments in connection with COVID-19 in particular have shown that the desire for robust chains is often greater than the desire for efficiency. But even after the effects of the Corona pandemic have faded, the shift to resilience is likely to be permanent. That is, the strategic focus will increasingly be on continuity, which means the maintenance of operational processes.

A significant part of the robustness and resilience of production processes and the supply chain is provided by the increase in digitalisation [20–23]. Modern technologies allow both more precise and accelerated processing of operations. On the one hand, this relates to communication between value-creation partners per se; on the other hand, with the help of these technologies, it is possible to carry out extensive data analyses that cannot be done by humans alone. Artificial intelligence can independently develop solutions to emerging problems based on dynamic models. Artificial intelligence, unlike conventional software, can learn from its own data accumulated over time as well as from data from other connected data sources. Forecasting, risk management and resilience are given a decisive boost by the use of artificial intelligence [24].

In response to all the needs and demands, the European project *knowlEdge - Towards Artificial Intelligence powered manufacturing services, processes, and products in an edge-to-cloud-knowledge continuum for humans [in-the-loop]* (2021–2023) has developed an architecture for an information technology platform that combines the needed requirements (www.knowlEdge-project.eu).

The article presents a case study approach in which the primary actor of analysis is a company from the dairy food industry. The study is a combination of theorizing and empirical qualitative analysis of the initial development phases, describing the application of artificial intelligence to operational planning and the envisioned impacts, both short and long-term. The *knowlEdge* project has started in January 2021, is scheduled to run for 3 years, and is currently in the phase of integrating components and implementing the use cases. Thus, it does not depict final results but contains expected outcomes based on performance indicators.

Before outlining the architecture and applying it to the use case, I first provide a background by discussing the benefits of artificial intelligence in the supply chain, then write about the characteristics of adaptive supply networks.

## Artificial intelligence in the supply chain

Artificial intelligence aims to imitate certain human abilities concerning learning and thinking. IT systems can independently find solutions to problems that arise using artificial intelligence. Dynamic models can be created based on collected data - i.e. experience. The data basis does not necessarily have to be the system’s own; unknown parameters from other data sources can also be included. Depending on the requirements, data can be found, extracted, summarised and analysed. This can then be used, for example, to determine probabilities of occurrence for events and to recognise behavioural patterns [4].

The idea of a learning organisation that uses technologies that perceive, recognise and also learn independently is reflected in the concept of cognitive manufacturing [25]. Analogously, we can speak of the associated supply chain of a cognitive supply chain [4].

Supply chain management is highly information-intensive, requiring an understanding of complex, interconnected decision-making processes that are critical to collaborative problem solving, particularly about joint demand planning and forecasting processes across diverse supply chain partners. Artificial intelligence, therefore, offers enormous benefits. It can improve efficient analytics and provide meaningful simulations and notifications. This will improve the efficiency of a supply chain, increase resource and energy efficiency, reduce emissions, significantly improve responsiveness, i.e. agility within the supply chain, and thus also ensure the security and optimal risk management of a supply network [26]. As such, the application of artificial intelligence is comprehensively transformative and has enormous potential for improving workflows and production processes.

## From rigid to adaptive supply chains

When data is collected and exchanged in the supply chain, different activities in the chain are coordinated with the partners and previous individual solutions ought to merge with others, the previous rigid and rather hierarchically organised supply chain is replaced by an adaptable and flexible structure [4, 27, 28]. In the evolving network, events and activities can be synchronised with each other [4]. This concerns predominantly production itself with its manifold processes, which is brought into line with the logistical material flow and the exchange of information necessary for this. Secondly, it also involves the employees of the companies throughout the entire network. Since the classical hierarchy is consciously abandoned, it is possible to react autonomously to unexpected events, disruptions or the like [29].

The most important characteristics of this approach are, for example, flexibility, speed and efficiency, as work is done in a way that bypasses hierarchies, whereby the hierarchy stands for rigidity and a lack of efficiency. Furthermore, the adaptable network can better adjust to customer wishes, which is best described by the term customization [30]. Greater decentralization strengthens situational awareness, which allows a variety of responses to events, changes and disruptions. With these characteristics, the prerequisites for the intelligent supply network are fulfilled.

The technical components of an agile supply network can learn [31–33]. This is to be understood in the context of cognitive technologies in general and artificial intelligence in particular. In cooperation with employees of a company or a supply network, recommendations for action can be derived, for example. The employees, who are embedded in various work process loops and who are also learning themselves, form a cognitive, learning organisation with artificial intelligence. Thus, a supply network has an evolutionary characteristic. This means that employees can flexibly adapt their respective work processes, which are embedded in the network in the broadest sense, and also change them at short notice.

Ideally, the adaptation also applies in the opposite sense, namely when employees support the data basis of the artificial intelligence based on their own experience, i.e. contribute experience and domain expertise in such a way that the respective algorithms experience a fundamental quality improvement. This is an improvement of the kind that artificial intelligence models cannot contribute solely based on their data collection.

**Fig. 1:**
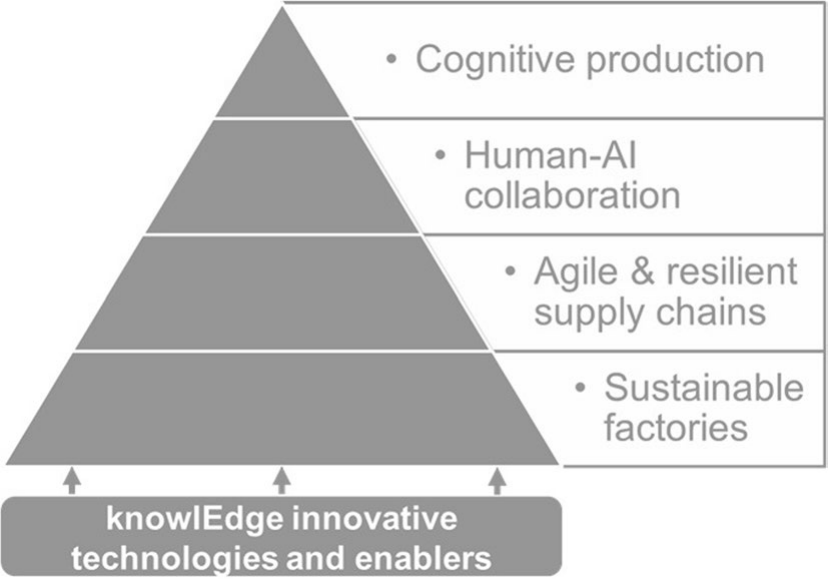
knowlEdge response to the needs of industrial supply chains

The aspiration to realise sustainable factories aims, on the one hand, to create significant and sustainable competitive gains through the intelligent synthesis of technologies, tools and methods. Artificial intelligence, as described, can help companies to operate successfully in an increasingly challenging environment where change seems unpredictable but is nonetheless continuous. The second aspect is focused on increasing resource and energy efficiency. Due to the increasing demand for goods, relative scarcity of resources and the desire to mitigate the effects of climate change, the introduction and diffusion of artificial intelligence tools is increasingly important. The goal of producing more with less is an important contribution to achieving environmental sustainability. The technical foundations of *knowlEdge* enable, for example, the production of zero-defect products with predictable quality. At the same time, planning processes can be optimised due to improved decision support. Both contribute to lower energy consumption and waste generation throughout the supply network.

Figure 1 highlights the major areas and improvements, which the *knowlEdge* project’s main product, an information technology platform, creates. It constitutes the foundation for the fundamental activities and processes in companies and across supply networks, helps to monitor material and information flows, and supports decision-making, both at operational and strategic levels of management [1, 34, 35].

## The knowlEdge platform

**Fig. 2:**
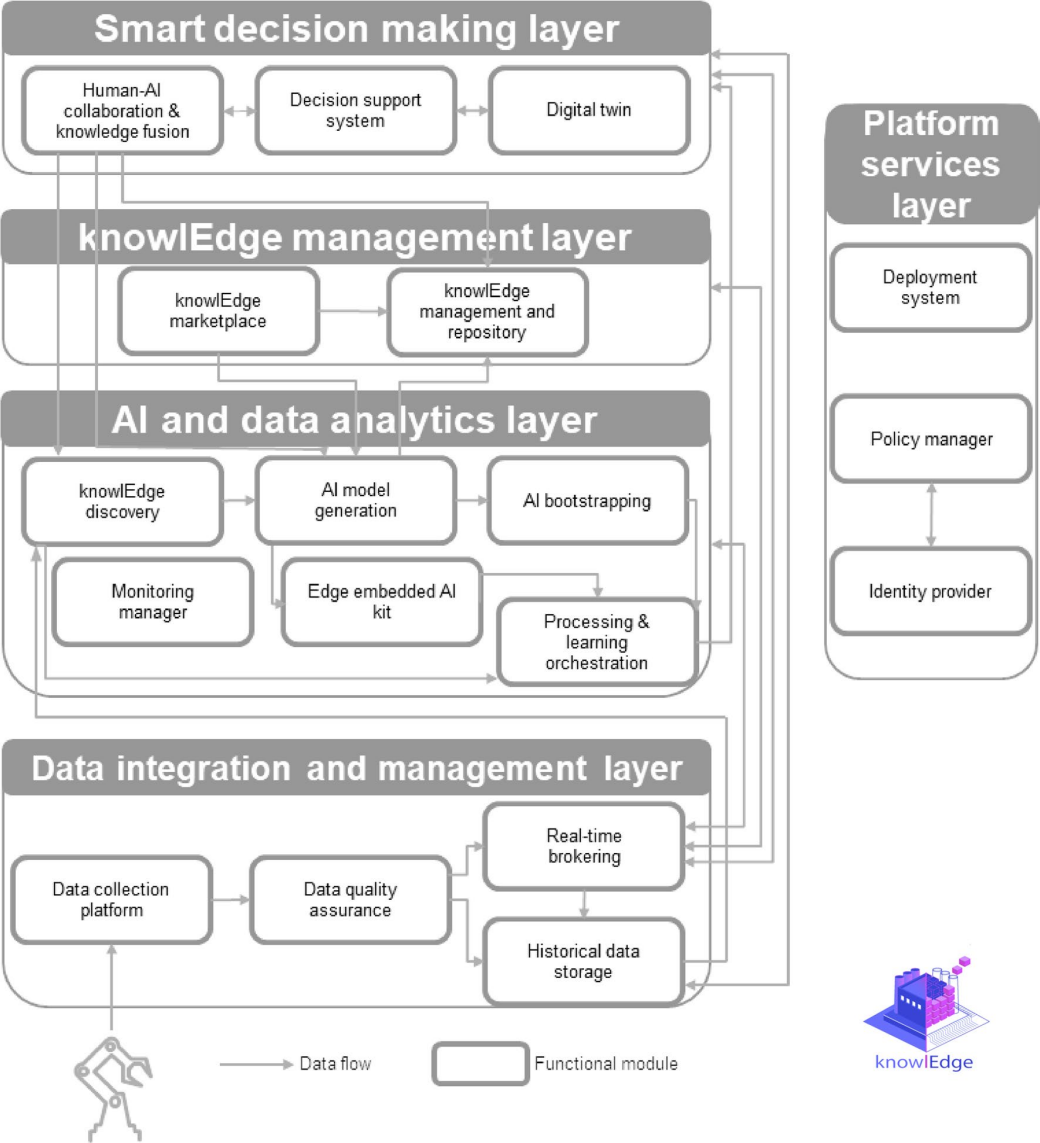
knowlEdge platform architecture (knowlEdge-project.eu)

The *knowlEdge* platform [36] was developed in response to the fact that artificial intelligence is one of the biggest trends in current industrial development and the resulting need for companies to adapt to remain competitive (Fig. 2). It is also important to consider that the barriers to the use of artificial intelligence, in general, are extraordinarily high. This includes the complexity of developing such algorithms, which is exacerbated by the lack of skilled labour. Therefore, the architecture is developing artificial intelligence solutions that are not only agile, but also collaborative, standardised and reusable. The requirement to be accountable and secure is also supported. In terms of the requirements of a supply network, it is equally indispensable to offer distributed and scalable solutions.

From a methodological perspective, *knowlEdge* integrates cognitive technologies from different domains, including artificial intelligence, distributed data analytics, embedded computing, internet-of-things, cyber-physical systems (CPS), software engineering, edge and cloud technologies, into a unified software architecture. This is particularly interesting and relevant where the combined integration of diverse functionalities is a challenge, which is especially the case in integrated supply chains. The unified architecture here addresses the problem of interfaces, which continues to cause breaks in the exchange of information and problems in countless companies, alliances and supply networks.

Developments in *knowlEdge* architecture include several main elements whose integration, as described, addresses the biggest challenges for supply chains. These elements include an intelligent decision-making layer that provides interfaces to users and offers the so-called human-in-the-loop aspects of the platform. These interfaces enable human knowledge, for example in the form of expertise on specific facts, routines, supply processes or the like, to be brought into the algorithm, to improve the data generated from the models, to understand analytical results via advanced user interfaces and to gain insights into processes.

Second, the architecture contains a management layer, which serves to semantically represent and store knowledge generated and consumed in all parts of the platform and various related services and to exchange/sell this knowledge. This is a feature, which can greatly accelerate the expansion and application of artificial intelligence routines across a supply network or indeed an entire industry. It can also lead to an entirely new business model for companies involved in training and subsequently selling adequate models.

Third, the platform includes an artificial intelligence and data analytics layer that covers the life cycle of an artificial intelligence algorithm from function development, model training, model evaluation and assessment, model deployment and model maintenance. Fourth, the architecture also incorporates a data integration and management layer that is responsible for ingesting data from the supply network into the platform. This can be data from a factory floor, manufacturing systems, warehouses or even vehicles, and from diverse and geographically dispersed sources. This module thus ensures the interoperability between different components which is indispensable, especially from the point of view of a supply network. The data can be provided from the IT side within a continuum of cloud, fog and edge.

As a fifth module, the architecture also includes a platform service layer that contains cross-platform functions and covers the provision of algorithms in the platform. In addition, this module contains components that ensure the security of the platform.

The combination of the technologies already mentioned at the beginning into a unified architecture prepares the basis for several important innovations. The architecture enables knowledge discovery and management, distributed and parallel computing, digital and multi-sided platforms as an infrastructure capability, data management and data administration, use of a computing continuum in the form of edge-to-cloud deployment capabilities, plus the ambition to create or enable human-centred design and human-computer interaction.

## Use case: applying the technology

During the development phase of the architecture, several use cases from different manufacturing and process industries were selected [37]. These cases indicate an obvious need for AI-based adaptations to address challenges. These challenges are related to process improvements, for example, energy and resource efficiency and waste reduction, and enabling and promoting human engagement (human-in-the-loop) in process chains. Other issues to be considered that slow down the digitalisation of manufacturing processes in general and supply networks include data quality issues, lack of IT infrastructures and secure frameworks for the use of such advanced support tools as artificial intelligence.

In the following, the focus will be on a company in the dairy food industry. The use case is appropriate because it shows typical problems that a supply chain brings with it and that can be addressed with the help of the AI solution and the integrated architecture. The designated company mainly processes or produces fresh milk and dairy products. As perishable products, the coordination of their production processes and their consequences for the supply chains becomes central. Both, robustness in terms of maintaining production and resilience to respond to unexpected disruptions are important. This is not only to be understood in the context of a general increase in efficiency or the avoidance of unnecessary waste. Coordination is also crucial for the sustainability of food systems.

The basic problem is optimal planning and scheduling of the supply chain, forecasting, and optimisation of production batches. Therefore, one objective is to develop a finite capacity planning tool, which supports planning the production flows considering the constraints and limits of the entire production flow, for example the production capacity of certain production lines. The approach will help improve production efficiency and reduce potential losses. Under the second objective, information will be extracted from various data streams (production data, warehouse data, product traceability, measurements in the workshop, etc.). This will allow for better prediction of requested volumes, optimisation of internal inventory management, reduction of raw material inventories, improvement of production processes, and reduction of waste. In addition, production and related logistics processes can be better coordinated [38].

The solution to the above challenges is based on the platform’s functions and consists of three scenarios. First, planning the production schedule; second, monitoring production; and third, adjusting the production process in real-time. Together, these scenarios cover the process of demand, forecasting, planning and monitoring the flow of production in a typical manufacturing company [37, 38].

*Production schedule planning* involves advanced analyses to identify constraints and prioritize orders in order to optimize production efficiency. By showing constraints between machines, production lines, and processes, the artificial intelligence system can help to identify potential bottlenecks and prioritize orders accordingly. This ensures that production runs with minimal downtime. By simulating the entire production process in the plant and taking into account the current inventory state of the warehouse, the system can provide statements about the need for raw materials and suggest possible rescheduling of the production schedule.
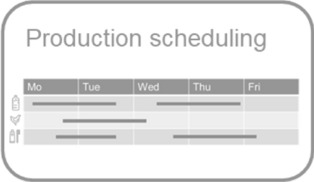


*Monitoring production* involves automated systems collecting data directly from sensors within the production facilities in real-time and integrating it from a wide range of sources, including data from mobile sources such as logistical data. This allows for a comprehensive and detailed picture of the production process to be generated in real-time. The system then uses this data to provide suggestions for decision-making, which can be based on both real-time data and historical data. By continuously collecting and evaluating real-time data, the system can identify deviations from the norm and provide suggestions for corrective action. Historical data can also be used to compare current performance with past performance.
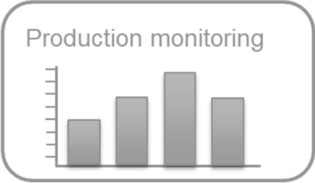


*Real-time adjustments to the production process* involves constantly monitoring and analyzing the production process. The artificial intelligence system can quickly detect any deviations and identify their causes and consequences for upstream and downstream activities. This allows for timely interventions to rectify any problems and minimize their impact on the production process. The use of historical data and real-time monitoring also allows the system to offer suggestions for possible decisions. This helps employees to make informed decisions quickly and efficiently, saving time and reducing the likelihood of errors. By allowing employees to intervene and improve the system’s database, the AI system can continuously learn and improve over time, making it even more effective at identifying and addressing production issues. Documenting any deviations and errors, as well as their remedies, is also essential in continuously expanding the database and improving the AI system.
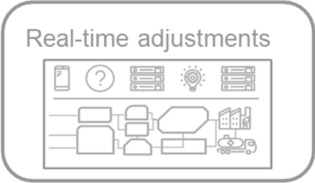


In terms of concrete and measurable improvements, several key performance indicators (KPI) were developed (Table 1). The Scheduling Hours metric aims to reduce the time spent on production planning or scheduling. The number of hours should be reduced by half on average. This is followed by the Response Time needed to create a new production plan based on real-time data, which should also be reduced by half. This KPI reflects both the time it takes to respond to a disruption or unexpected event in the supply chain and a robust supply chain design. The simulation capabilities of the solution aim to increase the Overall Equipment Effectiveness (OEE) by two to five percent when compared with data based on historical performance. OEE refers to the measurement of the actual availability of production equipment, its utilisation and quality. This KPI is therefore significant because it aims to show the assurance of reliability, availability and end-to-end process stability. Conversely, underutilisation and low plant efficiency can have an impact on the stability of value chains. The metric is, therefore, a contribution to the transparency of supply chain utilisation and robustness. Further, the Productivity metric refers to the reduction in the number of man-hours required to produce one ton of product. Specifically, man-hours are to be reduced by two to five per cent. Closely related to OEE is Downtime. Here, the goal is to reduce downtime and set-up times per ton of production by ten percent in one week. The objective of reducing downtime aims to improve the robustness of the supply chain. At the same time, Scrap should also be reduced by ten per cent per ton of production per week. Overall, the Forecasting Accuracy, i.e., the reduction of the percentage error rate in the prediction compared to the prediction of the actual production data, is to be reduced by ten per cent. A high level of forecasting accuracy indicates that a supply chain is robust and able to effectively anticipate demand fluctuations [39].

**Table 1:** KPI’s in use case [39]

KPI	Objective	Target%	Evaluation
Scheduling hours	Reduce time spent on production planning or scheduling	−50	Average of weekly hours for production planning
Response time	Reduce time to generate new production plan based on real-time data	−50	Timestamp of a change or deviation event and the delivery time of a revised plan
Overall Equipment Efficiency (OEE)	Improve the OEE of the production line	2 to 5	Apply AI solution based on historical production data
Productivity	Reduce the number of man-hours required to produce one ton of the product	−2 to −5	Use of data on production and hours worked on the production line from historical data
Downtime	Reduce downtime and set-up time	−10	Duration of refitting and set-up work per ton produced in one week
Scrap	Reduce the amount of scrap	−10	Scrap per ton produced in one week
Forecasting accuracy	Reduce the error rate in forecast	−10	Compare the forecast with the actual production data

## Discussion

Based on the above architecture and the outlined scenarios for the application of artificial intelligence, the first components for the case study have already been developed and integrated. By the end of the project (12/2023), the understanding of the platform's capability in the company and the possibilities for interaction between the software solutions and the human operators will increase. This will ensure effective use.

As a result of the work, the company expects to be faster in the production planning phase and thus improve the collaboration for its upstream suppliers and the service level for its downstream customers. Through better forecasting, it is also expected that inventory management and other operational activities will be supported in such a way that the company can better adapt to the increasing variability of the market. Responsiveness is expected to be integrated into the production structure. Remaining manual tasks in planning will be significantly facilitated [38].

Since the solutions can be applied to a wide range of areas, including pre-planning, actual production planning and production execution, the platform is also relevant to a wide range of manufacturing companies that have similar challenges and need to respond to them similarly. Moreover, the concept of generative planning combines artificial intelligence and human creativity to deliver products or services at an accelerated rate [40]. The human-in-the-loop aspect, as considered in the production planning use case, is an element of collaborative planning. This is an important factor in the agility of the whole complex because human engagement coincides with a greater degree of autonomy. Decision-making in this use context is not centralized but up to the discretion of the human operator. In unforeseen situations and circumstances, the system can react and is not paralysed due to a rigid structure, both in terms of traditional information technology solutions and hierarchical management approaches. As a result, we can expect more resilient supply networks.

Overall, the platform is a systemic solution, not just an incremental adaptation. It can increase the performance of company actors in entire supply chains, provided that appropriate algorithms and suitable data flow infrastructures exist at the relevant interfaces. This increase leads to greater adaptability and flexibility. This leap in performance can also be attributed to individual nodes in the supply network, i.e. from individual devices that serve as integrated data sources to employees who use their wealth of experience to complement artificial intelligence.

While it is almost obvious that artificial intelligence can revolutionise practices in the industrial supply chain, not all organisations are capable or ready to adopt this technology. Important areas where challenges exist include strategy and management, products and services, competencies and capabilities, collaboration, internal processes, data use and readiness for technology adoption [41]. From a strategic perspective, a company’s management must understand the benefits of using AI in its business activities. For example, relevant expertise and skills should be in place, even if the goal of software developers and designers is to develop more self-explanatory solutions. Data requirements also need to be understood and confidentiality and compliance requirements defined, both in terms of business needs and legal requirements. In addition, organisations would do well to define a roadmap for the adoption and use of AI solutions and understand how new solutions can be integrated with existing processes and legacy technology.

## Conclusion

The *knowlEdge* innovations lead to a new generation of AI-based services and can transform the way supply chains are managed. The industry can adopt agile and flexible strategies using its integrated supply chains. This allows to rapidly react to changing customer needs while optimising processes and quality control. A learning environment ensures that future deviations in planned processes can be addressed better.

In this context, it is conceivable that rigid organizational management structures will be complemented or even increasingly replaced with decentralized decision-making. Thus, the changes that accompany the introduction of advanced information technology and artificial intelligence will have an impact on the understanding of future management, both strategic and operational.

Learning is the central theme of the cognitive supply chain. Learning stands for evolution and for the ability to adapt to changing conditions. The indeterminacy of the future is not a problem for a learning cognitive system. The architecture of the *knowlEdge* platform, in which humans can form a strong alliance with technology, will therefore also be of particular importance concerning the adaptive capacity, resilience and sustainability performance in the supply network.

The possibilities for human engagement in a supply chain shaped by cognitive technologies have only been touched upon here. Future research will have to address how knowledge in the form of experience and domain expertise can be captured in different work environments and contexts.

## Data Availability

Supporting project deliverables are available at the Community Research and Development Information Service (CORDIS) of the European Commission.

## References

[1] Bowersox DJ, Closs DJ, Cooper MB, Bowersox JC. Supply chain logistics management. 4th ed. New York, NY, USA: McGraw-Hill; 2016.

[2] Christopher M. Logistics and supply chain management: strategies for reducing cost and improving service. 2nd ed. Harlow, UK: Prentice-Hall; 1998.

[3] Porter ME. Competitive advantage. New York: Free Press; 1985.

[4] Werner H. Supply chain management: grundlagen, strategien, instrumente and controlling. 7th ed. Wiesbaden: Springer; 2020.

[5] Ketchen DJ Jr, Hult GTM. Bridging organization theory and supply chain management: the case of best value supply chains. J Oper Manage. 2007;25:573–80.

[6] Lii P, Kuo FI. Innovation-oriented supply chain integration for combined competitiveness and firm performance. Int J Prod Eco. 2016;60:142–55.

[7] McDougall N, Wagner B, MacBryde J. Leveraging competitiveness from sustainable operations: frameworks to understand the dynamic capabilities needed to realise nrbv supply chain strategies. Supply Chain Manag. 2022;27:12–29.

[8] Di Serio CSLC, de Vicente Bittar A. Impact of supply chain on the competitiveness of the automotive industry. RAUSP Manag J. 2019;54:205–25.

[9] Butt AS, Sohal A, Prajogo D. Personal relationships and the loyalty in supply chain. J Devel Areas. 2019;53:239–46.

[10] Del Vecchio C, Paschalidis IC. Enforcing service-level constraints in supply chains with assembly operations. IEEE Trans Automatic Control. 2006;51:2000–5.

[11] Khan I, Lim H, Jemai J, Sarkar B. Effect of electrical energy on the manufacturing setup cost reduction, transportation discounts, and process quality improvement in a two-echelon supply chain management under a service-level constraint. Energies. 2019;12:3733.

[12] Mirkovski K, Davison RM, Martinsons MG. The effects of trust and distrust on ict-enabled information sharing in supply chains : evidence from small- and medium-sized enterprises in two developing economies. Int J Logist Manag. 2019;30:892–926.

[13] Qian C, Seuring S, Wagner R, Dion PA. Personal and organizational level relationships in relational exchanges in supply chains—a bottom-up model. Supply Chain Manag An Int J. 2020;26:32–47.

[14] Sarkar M, Chung BD. Flexible work-in-process production system in supply chain management under quality improvement. Int J Prod Res. 2019;58:3821–38.

[15] Wang B, Kang Y, Childerhouse P, Huo B. Interpersonal and inter-organizational relationship drivers of supply chain integration. Ind Manag Data Syst. 2018;118:1170–91.

[16] Malik M, Ghaderi H, Andargoli A. A resource orchestration view of supply chain traceability and transparency bundles for competitive advantage. Bus Strat Environ. 2021;30:3866–81.

[17] McGrath P, McCarthy L, Marshall D, Rehme J. Tools and technologies of transparency in sustainable global supply chains. California Manage Rev. 2021;64:67–89.

[18] Zhou D. An empirical study of the role of postponement application in reducing supply chain complexity. IEEE International Engineering Management Conference. 2002. 1; 448–53. 10.1109/IEMC.2002.1038474

[19] Aldrighetti R, Battini D, Ivanov D, Zennaro I. Costs of resilience and disruptions in supply chain network design models: a review and future research directions. Int J Prod Eco. 2021;235: 108103.

[20] Michel-Villarreal R, Vilalta-Perdomo EL, Canavari M, Hingley M. Resilience and digitalization in short food supply chains: a case study approach. Sustainability. 2021;13:5913.

[21] Naz F, Kumar A, Majumdar A, Agrawal R. Is artificial intelligence an enabler of supply chain resiliency post covid-19? an exploratory state-of-the-art review for future research. Operations Management Research: Advancing Practice through Theory; 2021.

[22] Sengupta T, Narayanamurthy G, Moser R, Pereira V, Bhattacharjee D. Disruptive technologies for achieving supply chain resilience in covid-19 era: an implementation case study of satellite imagery and blockchain technologies in fish supply chain. Information Systems Frontiers: A Journal of Research and Innovation, 2021. 1–17. 10.1007/s10796-021-10228-3 PMC8639852 34876876

[23] Trabucco M, De Giovanni PAW. Achieving resilience and business sustainability during covid-19: the role of lean supply chain practices and digitalization. Sustainability. 2021;13:12369.

[24] Dolgui, A., Bernard, A., Lemoine, D., von Cieminski, G., Romero, D. (eds.): Advances in Production Management Systems. Artificial Intelligence for Sustainable and Resilient Production Systems. IFIP WG 5.7 International Conference, APMS 2021, Nantes, France, September 5-9, 2021, Proceedings, Parts I-V. Springer, Cham (2021). 10.1007/978-3-030-85874-2

[25] Zheng P, Xia L, Li C, Li X, Liu B. Towards self-x cognitive manufacturing network: an industrial knowledge graph-based multi-agent reinforcement learning approach. J Manuf Syst. 2021;61:16–26.

[26] Min H. Artificial intelligence in supply chain management: theory and applications. Int J Logist Res Appl. 2019;13:13–39.

[27] Bak O. Understanding the stimuli, scope, and impact of organizational transformation: the context of ebusiness technologies in supply chains. Strategic Change. 2021;30:443–52.

[28] Zhang J, Xu J, Liu, Y. Complex adaptive supply chain network: the state of the art. In: 2009 Chinese Control and Decision Conference. 2009. pp. 5643–5647 . 10.1109/CCDC.2009.5195204.

[29] Snowdon AW, Saunders M. Covid-19, workforce autonomy and the health supply chain. Healthcare Quarterly. 2021;24:15–26. 34297659 10.12927/hcq.2021.26551

[30] Maozhu J, Wang H, Zhang Q, Zeng Y. Supply chain optimization based on chain management and mass customization. Inform Syst e-Business Manage. 2020;18:647–64.

[31] Iarovyi S, Lastra JLM, Haber R, del Toro R. From artificial cognitive systems and open architectures to cognitive manufacturing systems. In: 2015 IEEE 13th International Conference on Industrial Informatics (INDIN). 2015. pp. 1225–1232 . 10.1109/INDIN.2015.7281910.

[32] Li S, Wang R, Zheng P, Wang L. Towards proactive human-robot collaboration: a foreseeable cognitive manufacturing paradigm. J Manuf Syst. 2021;60:547–52.

[33] Mavrikios D, Papakostas N, Mourtzis D, Chryssolouris G. On industrial learning and training for the factories of the future: a conceptual, cognitive and technology framework. J Intell Manuf. 2013;24:473–85.

[34] Gattorna J. Dynamic supply chains: how to design, build and manage people-centric value networks. 3rd ed. Harlow, UK: Pearson Education Limited/FT Publishing International; 2015.

[35] Schulte C. Logistik: Wege zur optimierung der supply chain. 6th ed. München: Vahlen Verlag; 2013.

[36] Anaya V. (ed.): D2.4 Vision, specification and system architecture. In: WP2 system engineering, specifications and external collaboration, deliverable of knowlEdge project consortium. knowlEdge consortium, www.knowledge-project.eu 2022.

[37] Pins D. (ed.): D2.1 User need analysis and scenario definition. In: WP2 system engineering, specifications and external collaboration, deliverable of knowlEdge project consortium. knowlEdge consortium, www.knowledge-project.eu 2022.

[38] Ziliotti L. and Musiari E. (eds.): D8.3 Parmalat demonstrator. In: WP8 applications of the AI services on the shop-floor, deliverable of knowlEdge project consortium. knowlEdge consortium, www.knowledge-project.eu 2022.

[39] Saari L. (ed.): D8.2 Final evaluation KPIs. In: WP8 applications of the AI services on the shop-floor, deliverable of knowlEdge project consortium. knowlEdge consortium, www.knowledge-project.eu 2022.

[40] Barrué C. (ed.): D2.3 Market radar and technology adaptations. In: WP2 system engineering, specifications and external collaboration, deliverable of knowlEdge project consortium. knowlEdge consortium, www.knowledge-project.eu 2022.

[41] Saari L. Kuusisto O. Pirttikangas S. AI maturity web tool helps organisations proceed with AI. VTT white paper, VTT technical research centre of Finland. 2019. 10.32040/Whitepaper.2019.AIMaturity.

